# The Association Between Visceral Adiposity Index and Worsening Renal Function in the Elderly

**DOI:** 10.3389/fnut.2022.861801

**Published:** 2022-03-24

**Authors:** Li Lei, Lei Dai, Qiuxia Zhang, Junyan Lu, Yongzhen Tang, Min Xiao, Guodong Li, Shaohua Yan, Xiaobo Li, Yejia Chen, Yaode Chen, Yun Li, Shengli An, Jiancheng Xiu

**Affiliations:** ^1^Department of Cardiology, Nanfang Hospital, Southern Medical University, Guangzhou, China; ^2^Department of Cardiology, Zengcheng Branch of Nanfang Hospital, Guangzhou, China; ^3^Department of Public Health Management, Zengcheng Xintang Hospital, Guangzhou, China; ^4^Department of Biostatistics, School of Public Health, Southern Medical University, Guangzhou, China

**Keywords:** visceral adiposity index, renal function, chronic kidney disease, elderly, cohort study

## Abstract

**Background:**

Visceral adiposity index (VAI) is an indicator of visceral fat accumulation and dysfunction. However, little is known about whether VAI is associated with worsening renal function (WRF) in the elderly. Therefore, our study aimed to explore the association between VAI and WRF among the elderly population.

**Methods:**

In total, 5,583 elderly participants (aged ≥ 65 years) who participated in the annual health checkups at least twice between January 2017 and July 2021 were enrolled and divided into four groups according to the VAI quartiles. The primary endpoint was incident chronic kidney disease (CKD), defined as incident estimated glomerular filtration rate (eGFR) < 60 ml/min/1.73 m^2^. The secondary endpoint was rapid kidney function decline (RKFD), defined as decline in eGFR of 40%. To evaluate the association between VAI and WRF, three Cox regression models were conducted, where VAI was treated as a continuous variable and a categorical variable (Q1 as reference), respectively. Subgroup analysis in participants with different baseline characteristics was also performed.

**Results:**

During a median of 2.46 year follow-up, 931 (16.68%) participants developed CKD. After fully adjusting for confounding factors, VAI was significantly associated with incident CKD (HR, 1.052; 95% CI: 1.029–1.076, *p* < 0.001), and RKFD (HR, 1.077; 95% CI: 1.041–1.114, *p* < 0.001). Moreover, compared to those with the lowest VAI quartiles, subjects with the highest quartiles had a higher risk of incident CKD (HR, 1.286; 95% CI: 1.033–1.601, *p* = 0.024), and RKFD (HR, 1.895; 95% CI: 1.086–3.307, *p* = 0.025). The risk of incident CKD also tended to increase with elevated VAI quartiles (all *p*-values for trend <0.05). This positive association remained consistent among participants with different genders, baseline weights, or kidney functions.

**Conclusion:**

In our study, elevated VAI was associated with increased risk of incident CKD and RKFD in the elderly population.

## Introduction

With about 697.5 million patients of all-stage chronic kidney disease (CKD) in 2017 (global prevalence of 9.1%) and over 2.5 million patients undergoing renal replacement therapy globally, worsening renal function (WRF) is associated with increased risk of cardiovascular diseases and other adverse health events (1–3). Increasing evidence showed that obesity is associated with higher risk of CKD, whereas few studies have explored whether the accumulation of visceral fat is the leading cause of this association (4, 5). Visceral adiposity index (VAI), calculating with waist circumference (WC), body mass index (BMI), triglycerides (TG), and high-density lipoprotein (HDL), is a reliable indicator of visceral fat accumulation and dysfunction (6, 7). Moreover, previous studies have showed that visceral obesity is associated with pro-inflammatory activity and insulin resistance, which are both risk factors of renal function deterioration (5, 8). Recently, results from some cross-sectional studies showed that VAI is associated with higher prevalence of CKD, whereas evidence from the longitudinal studies is still limited, especially among the elderly (9–11). Therefore, the aim of this study was to explore the association between VAI and WRF in the elderly based on a retrospective cohort from southern China.

## Materials and Methods

### Study Design and Population

This retrospective study was conducted based on 6,543 elderly residents (aged ≥ 65 years) from 37 communities or villages of Guangzhou, Guangdong who participated in the annual health checkup provided by the Chinese National Basic Public Health Service project at least twice between January 2017 and July 2021. In this current analysis, we excluded participants with baseline CKD (*n* = 941) and those without sufficient baseline data to calculate VAI (*n* = 19). Finally, 5,583 participants were included. This study was performed in accordance with the Declaration of Helsinki and was approved by the Ethics Committee of the Nanfang Hospital (NFEC-2021-083). Data of the residents’ first annual health checkup participation will be considered as the baseline. After enrollment, all participants were required to attend the annual health checkup during each of the following years. During each annual health checkup, similar contents will be provided and results were saved in the personal electronic health records.

### Study Endpoints and Major Definitions

The primary endpoint of this study was incident CKD, defined as incident estimated glomerular filtration rate (eGFR) of less than 60 ml/min/1.73 m^2^ (12). The secondary endpoint was rapid kidney function decline (RKFD), defined as decline in eGFR of 40% during follow-up period (12). eGFR was calculated using the Modification of Diet in Renal Disease (MDRD) equation (13). VAI was calculated using the following sex-specific equations: men: [WC/(39.68 + (1.88 × BMI))] × (TG/1.03) × (1.31/HDL); women: [WC/(36.58 + (1.89 × BMI))] × (TG/0.81) × (1.52/HDL). The unit of WC was centimeter, whereas the unit of TG and HDL was mmol/L (6). BMI was calculated as weight (kg)/height (m)^2^. Diabetes was defined as undergoing glucose-lowering therapy or fasting glucose ≥ 7 mmol/L (14). Hypertension was defined as systolic blood pressure (SBP) ≥ 140 mmHg or diastolic blood pressure (DBP) ≥ 90 mmHg or having hypertensive treatment (12).

### Data Collection

The collected data included demographic information, smoking history, alcohol use history, exercise frequency, anthropometric measurements, medications, and laboratory assays. All data were collected through electronic health records obtained from the regional chronic disease management platform.

### Statistical Analysis

All the included participants were divided into four groups according to the quartiles of VAI. For normally and non-normally distributed continuous variables, data were expressed as mean ± SD and median + interquartile range, respectively. For categorical variables, data were expressed as percentage. Baseline differences between VAI quartile groups were compared through ANOVA for continuous variables or chi-square test for categorical variables.

To evaluate the association between VAI and WRF, three Cox regression models were conducted. The first one was a crude model without adjustment. The second one was adjusted for well-accepted risk factors for WRF reported by previous study (15) including age, gender, diabetes mellitus, baseline SBP, DBP, and eGFR. The third one was adjusted for all the variables in the second one, plus lifestyles and medications including exercise daily, drinking daily, smoking, hypertensive treatment, and glucose-lowering treatment. In these models, VAI was treated as a continuous variable and a categorical variable (Q1 as reference), respectively. Linear trend was evaluated through Cox regression model where VAI quartile was entered as ordinal variable and through adjusted restricted cubic spline (RCS). Moreover, to further explore the association between VAI and WRF among different kinds of people, we have also conducted subgroup analysis in different genders, baseline kidney functions, and BMIs. The *p* value < 0.05 was considered statistically significant. All analyses were conducted with R software (version 4.1.0; R Foundation for Statistical Computing, Vienna, Austria) and SPSS (version 26.0).

## Results

### Baseline Characteristics

Baseline characteristics of the entire cohort and participants stratified by the VAI quartiles are displayed in Table 1. Generally, the mean age of the entire cohort was 70.69 ± 5.38 years, and 40.53% of the participants were male. Patients with higher VAI tended to have higher heart rates, blood pressures, BMI, and waist. VAI was also associated with hypertension and diabetes and inversely associated with male gender and baseline eGFR. In addition, significant differences were also identified in smoking habits, drinking habits, and lipid profiles between VAI quartiles.

**TABLE 1:** Baseline characteristics of study participants stratified by visceral adiposity index quartiles.

	Overall	Q1 (≤1.052)	Q2 (1.052– ≤1.745)	Q3 (1.745– ≤2.890)	Q4 (>2.890)	*p-*value
N	5,583	1,396	1,396	1,395	1,396	
Age, years	70.69 ± 5.38	70.87 ± 5.51	70.75 ± 5.43	70.77 ± 5.41	70.38 ± 5.16	0.085
Male, n (%)	2,263 (40.53)	871 (62.39)	568 (40.69)	466 (33.41)	358 (25.64)	<0.001
Heart rate, bpm	76.93 ± 12.22	75.62 ± 11.99	76.24 ± 12.19	77.42 ± 12.21	78.44 ± 12.32	<0.001
SBP, mmHg	146.89 ± 19.53	143.22 ± 19.20	146.66 ± 19.62	147.99 ± 19.12	149.67 ± 19.62	<0.001
DBP, mmHg	81.62 ± 11.67	80.20 ± 11.80	81.43 ± 11.92	82.04 ± 11.63	82.81 ± 11.16	<0.001
BMI, kg/m^2^	24.06 ± 3.57	22.36 ± 3.39	23.90 ± 3.41	24.69 ± 3.51	25.30 ± 3.29	<0.001
Waist, cm	85.54 ± 9.51	80.36 ± 9.19	84.99 ± 8.90	87.43 ± 8.85	89.38 ± 8.62	<0.001
Hypertension, n (%)	3,984 (71.39)	894 (64.09)	967 (69.27)	1,044 (74.84)	1,079 (77.35)	<0.001
Diabetes, n (%)	820 (14.69)	127 (9.10)	175 (12.54)	216 (15.48)	302 (21.63)	<0.001
Smoking, n (%)	706 (12.65)	244 (17.48)	195 (13.97)	154 (11.04)	113 (8.09)	<0.001
Drinking Daily, n (%)	252 (4.51)	98 (7.02)	64 (4.58)	53 (3.80)	37 (2.65)	<0.001
Exercise Daily, n (%)	3,123 (55.94)	783 (56.09)	786 (56.30)	771 (55.27)	783 (56.09)	0.949
Fasting glucose, mmol/L	4.80 [4.26, 5.51]	4.70 [4.21, 5.27]	4.70 [4.20, 5.32]	4.83 [4.27, 5.58]	5.00 [4.39, 5.92]	<0.001
Baseline eGFR	84.20 [73.77, 96.56]	85.18 [75.31, 97.50]	84.26 [73.65, 96.19]	83.79 [73.89, 95.94]	83.41 [73.05, 96.35]	0.027
Total cholesterol, mmol/L	5.48 [4.79, 6.20]	5.22 [4.57, 5.93]	5.44 [4.75, 6.11]	5.61 [4.96, 6.36]	5.64 [4.94, 6.39]	<0.001
Triglyceride, mmol/L	1.38 [0.99, 2.00]	0.81 [0.67, 0.96]	1.18 [1.03, 1.35]	1.65 [1.42, 1.89]	2.62 [2.14, 3.39]	<0.001
LDL-C, mmol/L	3.34 [2.76, 3.93]	3.00 [2.46, 3.57]	3.34 [2.80, 3.93]	3.58 [3.02, 4.14]	3.44 [2.86, 4.01]	<0.001
HDL-C, mmol/L	1.34 [1.13, 1.58]	1.66 [1.44, 1.93]	1.42 [1.24, 1.61]	1.29 [1.13, 1.45]	1.08 [0.95, 1.22]	<0.001
Hypertensive treatment, n (%)	1,604 (32.52)	330 (25.70)	368 (29.58)	428 (35.55)	478 (39.80)	<0.001
Glucose-lowering treatment, n (%)	551 (10.80)	92 (6.98)	109 (8.50)	147 (11.70)	203 (16.28)	<0.001

*SBP, systolic blood pressure; DBP, diastolic blood pressure; BMI, body mass index; eGFR, estimated glomerular filtration rate; LDL-C, low-density lipoprotein cholesterol; and HDL-C, high-density lipoprotein cholesterol.*

### Association Between Visceral Adiposity Index and Worsening Renal Function

During the median follow-up period of 2.46 [1.29, 3.34] years, 931 (16.68%) participants developed CKD in the entire cohort. The association between VAI and WRF is displayed in Table 2. Compared with the 1st VAI quartiles, the 2nd, 3rd, and 4th VAI quartiles all seemed to be associated with a higher risk of incident CKD in the crude model. Furthermore, after adjusting for confounding factors, subjects with the highest quartiles of VAI still tended to have a higher risk of incident CKD (HR, 1.286; 95% CI: 1.033–1.601, *p* = 0.024), and RKFD (HR, 1.895; 95% CI: 1.086–3.307, *p* = 0.025), compared to those with the lowest quartiles. The risk of incident CKD and RKFD also tended to increase with elevated VAI quartiles (all *p* for trend <0.05). In addition, when treating VAI as a continuous variable, elevated VAI was associated with increased WRF risk with or without adjustment for confounding factors.

**TABLE 2:** Association between visceral adiposity index and worsening renal function.

		Crude model		Adjusted model 1^a^		Adjusted model 2^b^	
	Event (%)	HR (95% CI)	*p-*value	HR (95% CI)	*p-*value	HR (95% CI)	*p-*value
**Incident chronic kidney disease**							
VAI as continuous variable	not available	1.053 (1.035–1.072)	<0.001	1.046 (1.024–1.068)	<0.001	1.052 (1.029–1.076)	<0.001
Q1	176 (12.61)	Reference		Reference		Reference	
Q2	224 (16.05)	1.262 (1.036–1.538)	0.021	1.014 (0.830–1.239)	0.894	1.041 (0.839–1.293)	0.714
Q3	251 (17.99)	1.435 (1.184–1.740)	<0.001	1.137 (0.933–1.385)	0.204	1.119 (0.898–1.395)	0.315
Q4	280 (20.06)	1.661 (1.375–2.005)	<0.001	1.300 (1.065–1.587)	0.010	1.286 (1.033–1.601)	0.024
*p* for trend		<0.001		0.003		0.017	
**Rapid kidney function decline**							
VAI as continuous variable	not available	1.084 (1.052–1.116)	<0.001	1.067 (1.032–1.104)	<0.001	1.077 (1.041–1.114)	<0.001
Q1	31 (2.22)	Reference		Reference		Reference	
Q2	37 (2.65)	1.140 (0.707–1.838)	0.590	1.260 (0.765–2.074)	0.365	1.194 (0.667–2.136)	0.551
Q3	47 (3.37)	1.449 (0.920–2.280)	0.109	1.413 (0.869–2.299)	0.163	1.373 (0.774–2.434)	0.278
Q4	60 (4.30)	1.973 (1.279–3.044)	0.002	1.736 (1.073–2.807)	0.025	1.895 (1.086–3.307)	0.025
*p* for trend		<0.001		0.020		0.015	

*^a^Adjusted for age, gender, diabetes mellitus, baseline systolic blood pressure, diastolic blood pressure, and eGFR. ^b^Adjusted for all the variables in model 1, plus exercise daily, drinking daily, smoking, hypertensive treatment, and glucose-lowering treatment.*

### Subgroup Analysis

**FIGURE 1:**
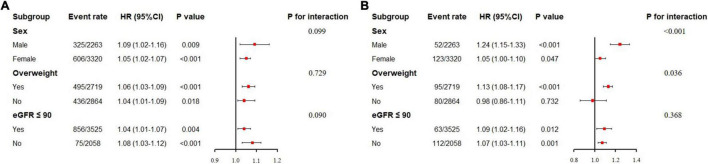
Subgroup analysis of association between visceral adiposity index and worsening renal function. eGFR: estimated glomerular filtration rate. **(A)** Subgroup analysis for incident chronic kidney disease. **(B)** Subgroup analysis for rapid kidney function decline. Visceral adiposity index here was treated as a continuous variable. For sex subgroup, the adjusted factors included age, baseline eGFR, systolic blood pressure, diastolic blood pressure, diabetes, hypertensive treatment, and glucose-lowering treatment. For overweight subgroup, the adjusted factors included age, sex, baseline eGFR, systolic blood pressure, diastolic blood pressure, diabetes, hypertensive treatment, and glucose-lowering treatment. For eGFR subgroup, the adjusted factors included age, sex, systolic blood pressure, diastolic blood pressure, diabetes, hypertensive treatment, and glucose-lowering treatment.

To further explore whether the association between VAI and WRF varied between different people, we also conducted subgroup analysis, and results are shown in Figure 1. Since RCS revealed a linear relationship between VAI and WRF (Supplementary Figures 1, 2), VAI was treated as a continuous variable in subgroup analysis. After adjusting for confounding factors, neither the sex, overweight, nor baseline kidney function altered the association between elevated VAI and increased risk of incident CKD (all *p*-values for interaction >0.05). However, for the secondary endpoint (RKFD), the HR of VAI was greater in male (HR, 1.24; 95% CI: 1.15–1.33, *p* < 0.001) as compared with female (HR, 1.05; 95% CI: 1.00–1.10, *p* = 0.047, *p* for interaction = 0.001). Moreover, VAI was significantly associated with RKFD in overweight participants (BMI ≥ 24), but not in those with BMI < 24.

## Discussion

Generally, our study is the first one to report the significant association between elevated VAI and increased risk of WRF in elderly people. This positive association between VAI and incident CKD remained consistent among participants with different genders, baseline weights, or kidney functions.

During the median follow-up period of 2.46 years, the occurrence rate of incident CKD in our study was 16.68% (*n* = 931). Another longitudinal study conducted by Bamba et al. (16) enrolled 15,159 general population without baseline medication or CKD. During the median 3.3-year follow-up for men and 3.2-year follow-up for women, the occurrence rate of incident CKD in their cohort was 7.62% and 6.51%, respectively (16). Comparing with the previous result, the occurrence rate of incident CKD in our cohort was higher. Older participants in our study (mean age: 70.69 ± 5.38 years) may be the main cause of this phenomenon.

A major finding of our study is that elevated VAI is associated with increased risk of WRF among the elderly, which is similar to the previous observations. Indeed, obesity and hyperlipidemia have long been reported to be associated with a higher risk of CKD (17). However, different adipose tissues have different functions, and not all adipose tissues are associated with metabolic disorders (18, 19). Several studies have tended to explore the association between visceral adiposity and WRF in different populations from our study. A longitudinal study conducted by Bamba et al. (16) enrolled 15,159 general population without baseline medication or CKD. Compared to the 1st quartile, the hazard ratios of incident CKD in the 4th quartile for men and women were 1.42 (95% CI: 1.06–1.90, *p* = 0.018) and 1.65 (95% CI: 1.12–2.46, *p* = 0.013), respectively (16). Another cohort including 10,699 hypertensive participants from the renal substudy of the China Stroke Primary Prevention Trial (CSPPT) reported that compared to those in the 1st quartile, patients in the 4th VAI quartile tended to have a higher risk of new-onset proteinuria (OR, 1.86; 95% CI: 1.12–3.11, *p* = 0.017), and progression of proteinuria (OR, 1.45; 95% CI: 1.08–1.95, *p* = 0.014), during a median follow-up period of 4.4 years (14). Similar results were also observed by Manabe et al. (20) among patients with CKD (20).

Rapid kidney function decline, defined as eGFR decline, is a suitable alternative endpoint for trials of kidney disease progression and has been proven to be associated with a higher risk of heart failure, myocardial infarction, and stroke (21, 22). In our study, we found that elevated VAI is also associated with increased risk of RKFD. However, in the subgroup analysis, this significant association was only observed in participants with BMI ≥ 24, but not in those with BMI < 24. In addition, elevated VAI seemed to be more dangerous in men rather than women. Future studies are needed to confirm these phenomena and to explore the potential causes.

Visceral adiposity index is an indicator for visceral fat accumulation and dysfunction (6). Although the potential mechanisms between visceral adiposity and kidney function deterioration still need further exploration, pro-inflammatory activity and insulin resistance may play an important role. Previous studies reported that visceral adipose tissue can release non-esterified fatty acids through lipolysis, which could damage podocytes and proximal tubular epithelial cells through inflammation and reactive oxygen species (ROS) and lead to insulin resistance (17, 23, 24).

Another point needed to be emphasized is that VAI, calculated with WC, BMI, TG, and HDL, does not directly reflect the visceral adipose tissue amount, nor does it allow quantifying different body tissues or different adipose tissue compartments. Future studies should adopt imaging techniques such as CT and MRI, which can provide non-invasive tissue assessment and characterization, to further explore the association between visceral adipose tissue and WRF (25).

There are still some limitations in the current study. First, the sample size of our study is relatively small. Second, the frequency of kidney function follow-up in our study was once a year, which may underestimate the effect of high VAI on WRF and cause insufficient accuracy in identifying endpoints. A more continuous observation of renal function is needed in future studies. Third, due to the insufficient data, some reported risk factors of WRF, such as proteinuria and albumin-to-creatinine ratio, were not adjusted in our analysis.

## Conclusion

In our study, we found that elevated VAI was positively associated with increased risk of incident CKD and RKFD in the elderly population. Larger and multicenter longitudinal studies or experimental trials testing VAI-lowering strategies are warranted to confirm this association in the future.

## Data Availability Statement

The raw data supporting the conclusions of this article will be made available by the authors, without undue reservation.

## Ethics Statement

The studies involving human participants were reviewed and approved by the Ethics Committee of Nanfang Hospital, Southern Medical University. The patients/participants provided their written informed consent to participate in this study.

## Author Contributions

LL, QZ, and JX: conception and design of the study. LL, LD, QZ, YT, MX, JL, GL, SY, XL, YJC, YDC, and YL: acquisition and analysis of data. LL, QZ, and SA: statistical analysis of data and interpretation. LL, LD, and QZ: preparation of the manuscript. SA and JX: revision of the manuscript. All authors read and approved the final version of the manuscript.

## Conflict of Interest

The authors declare that the research was conducted in the absence of any commercial or financial relationships that could be construed as a potential conflict of interest.

## Publisher’s Note

All claims expressed in this article are solely those of the authors and do not necessarily represent those of their affiliated organizations, or those of the publisher, the editors and the reviewers. Any product that may be evaluated in this article, or claim that may be made by its manufacturer, is not guaranteed or endorsed by the publisher.
