# Supplementary material for: The Association Between Visceral Adiposity Index and Worsening Renal Function in the Elderly
Source: Front Nutr. 2022 Mar 24;9:861801. doi: 10.3389/fnut.2022.861801 (PMC8987107; doi:10.3389/fnut.2022.861801)
Supplement: Supplementary file 1 [file Data_Sheet_1.docx]

Supplementary Material

**Supplementary Figure 1. Adjusted restricted cubic spline of the association between visceral adiposity index and incident chronic kidney disease.**

**
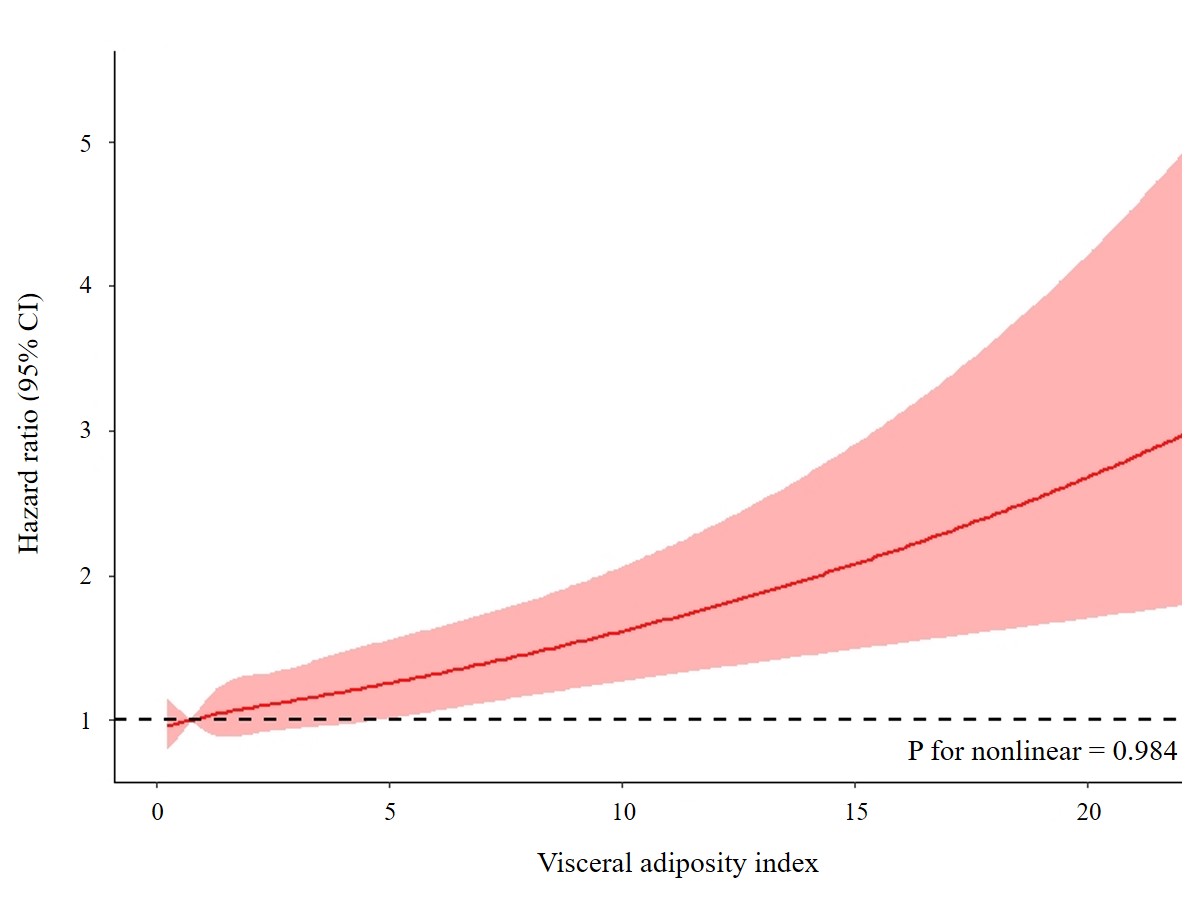
**

Adjusted for age, gender, diabetes mellitus, baseline systolic blood pressure, diastolic blood pressure, eGFR, exercise daily, drinking daily, smoking, hypertensive treatment and glucose-lowering treatment.

**Supplementary Figure 2. Adjusted restricted cubic spline of the association between visceral adiposity index and rapid kidney function decline.**

**
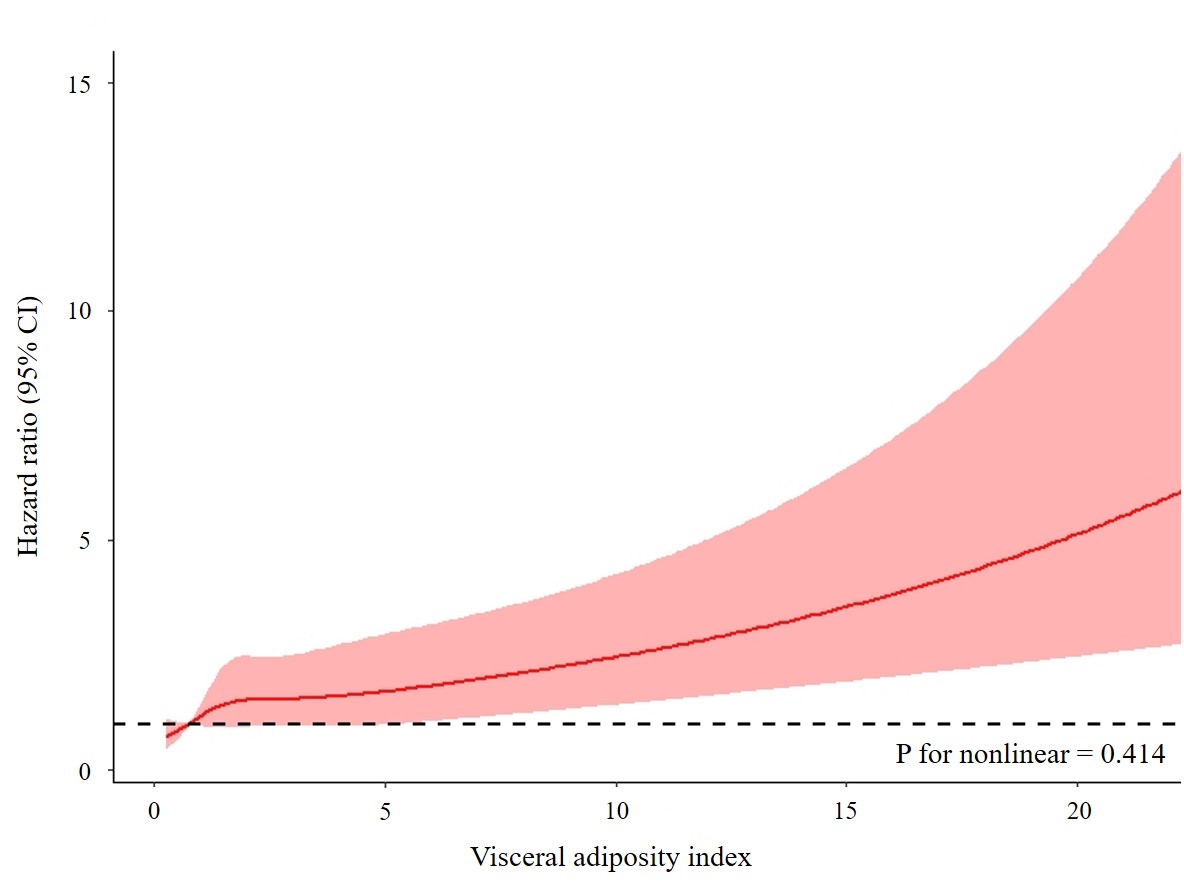
**

Adjusted for age, gender, diabetes mellitus, baseline systolic blood pressure, diastolic blood pressure, eGFR, exercise daily, drinking daily, smoking, hypertensive treatment and glucose-lowering treatment.
